# Supplementary material for: Rice TCD8 Encoding a Multi-Domain GTPase Is Crucial for Chloroplast Development of Early Leaf Stage at Low Temperatures
Source: Biology (Basel). 2022 Nov 29;11(12):1738. doi: 10.3390/biology11121738 (PMC9774597; doi:10.3390/biology11121738)
Supplement: Supplementary file 1 [file biology-11-01738-s001.zip › biology-2012452-supplementary.pptx]

## Slide 1
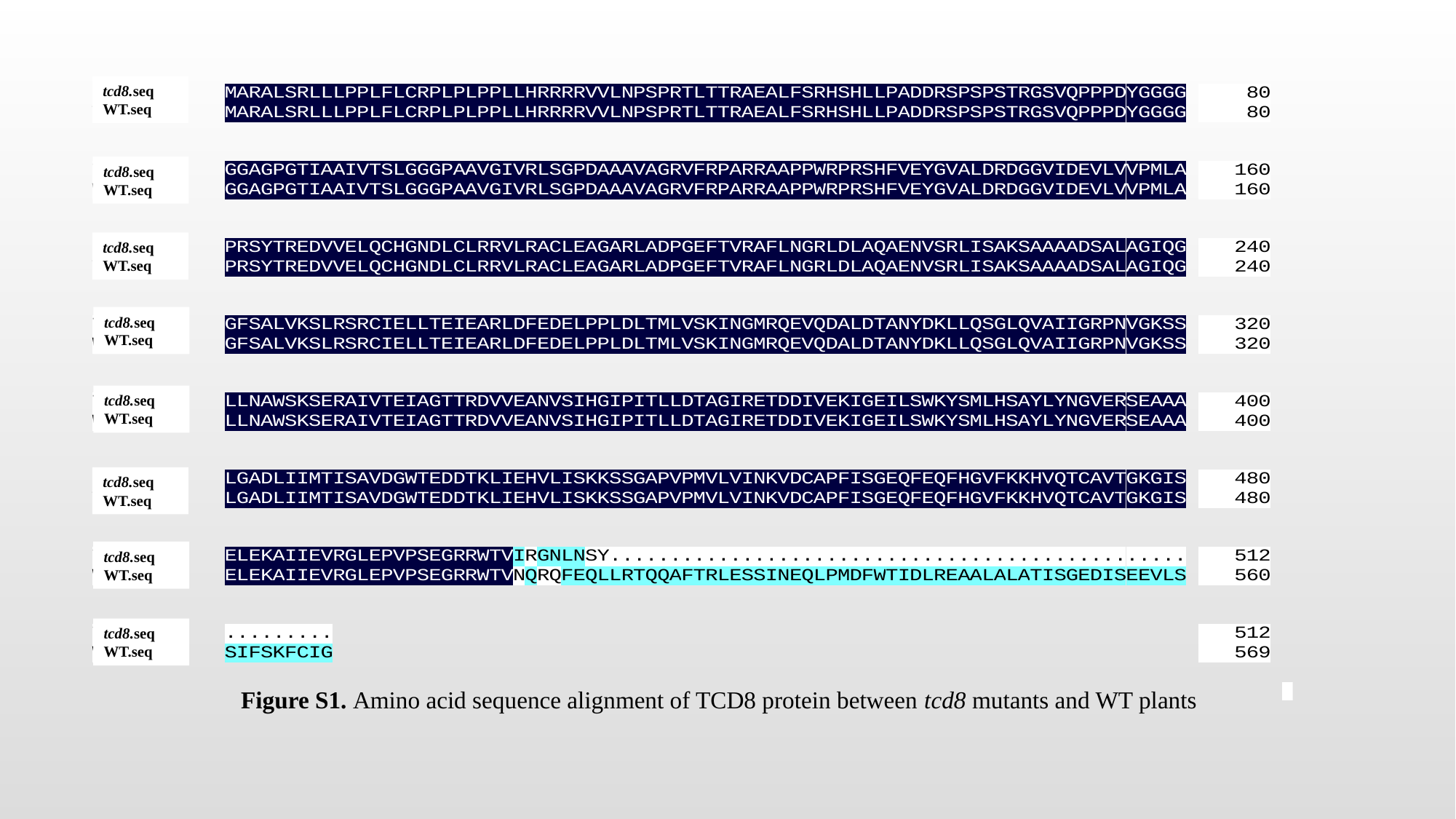

tcd8.seq
WT.seq
tcd8.seq
WT.seq
tcd8.seq
WT.seq
tcd8.seq
WT.seq
tcd8.seq
WT.seq
tcd8.seq
WT.seq
tcd8.seq
WT.seq
tcd8.seq
WT.seq
Figure S1. Amino acid sequence alignment of TCD8 protein between tcd8 mutants and WT plants

## Slide 2
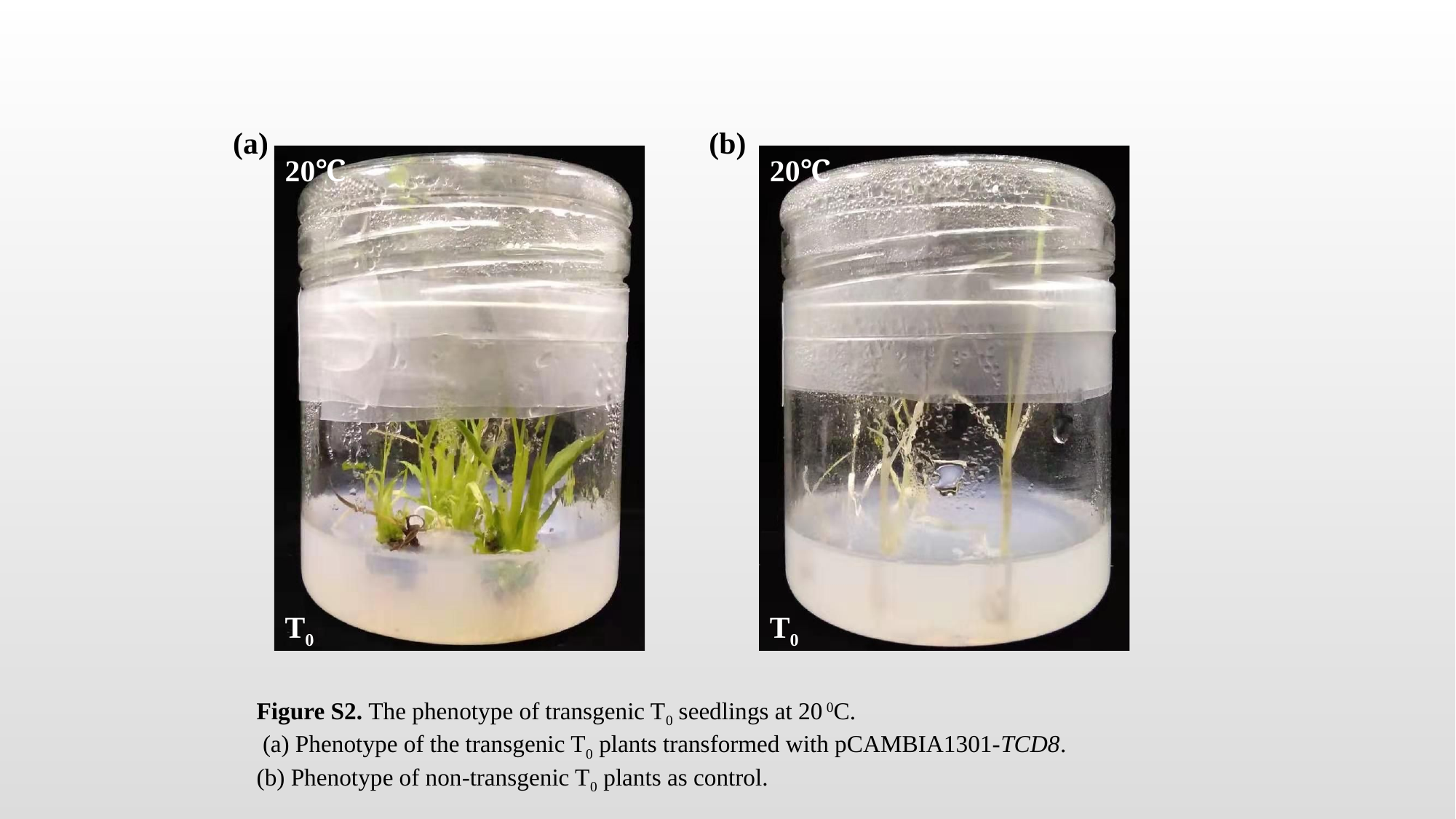

(a)
(b)
20℃
T0
20℃
T0
Figure S2. The phenotype of transgenic T0 seedlings at 20 0C.
 (a) Phenotype of the transgenic T0 plants transformed with pCAMBIA1301-TCD8. (b) Phenotype of non-transgenic T0 plants as control.

## Slide 3
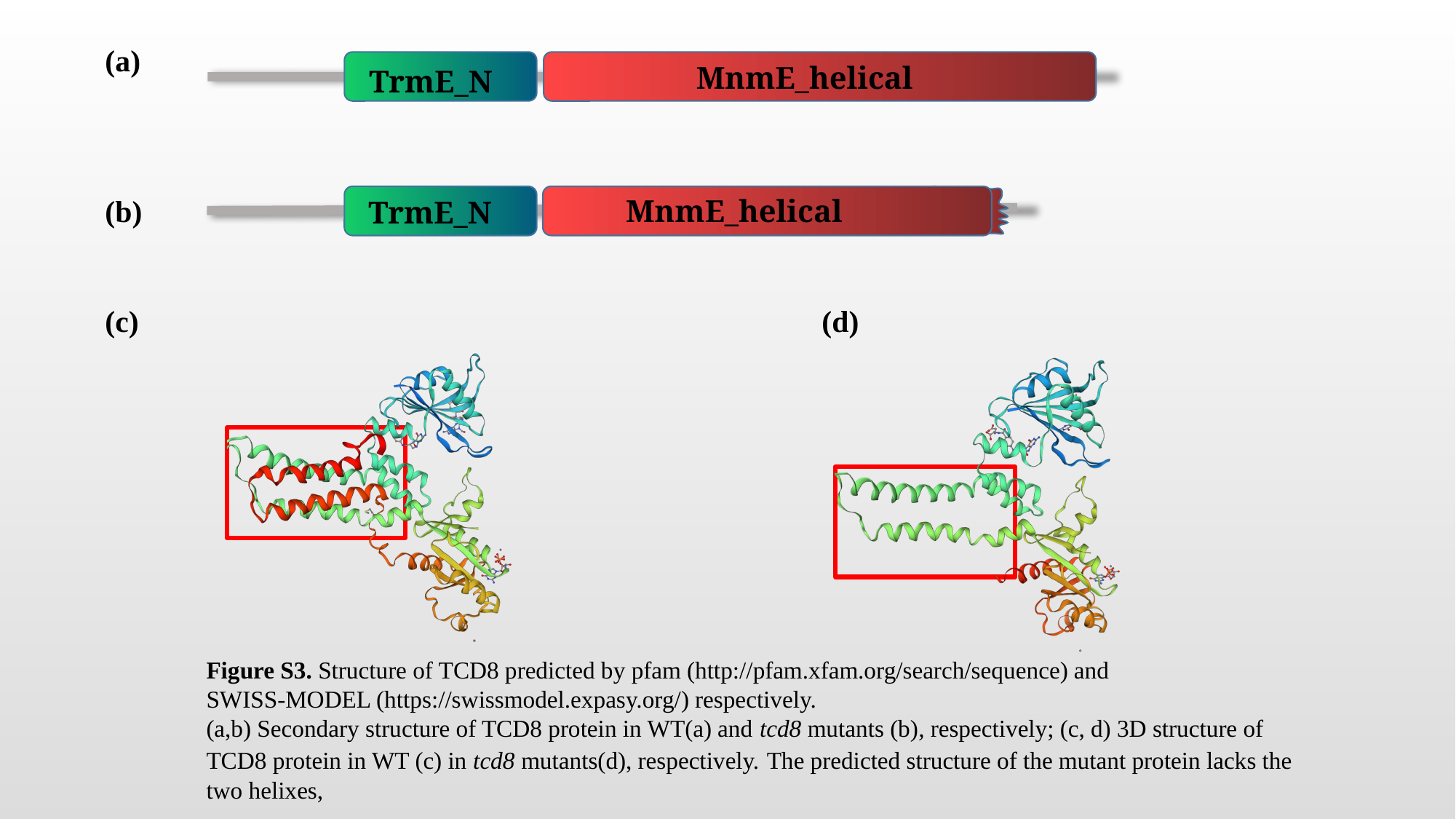

(a)
MnmE_helical
TrmE_N
(b)
TrmE_N
MnmE_helical
(c)
(d)
Figure S3. Structure of TCD8 predicted by pfam (http://pfam.xfam.org/search/sequence) and
SWISS-MODEL (https://swissmodel.expasy.org/) respectively.
(a,b) Secondary structure of TCD8 protein in WT(a) and tcd8 mutants (b), respectively; (c, d) 3D structure of TCD8 protein in WT (c) in tcd8 mutants(d), respectively. The predicted structure of the mutant protein lacks the two helixes,

## Slide 4
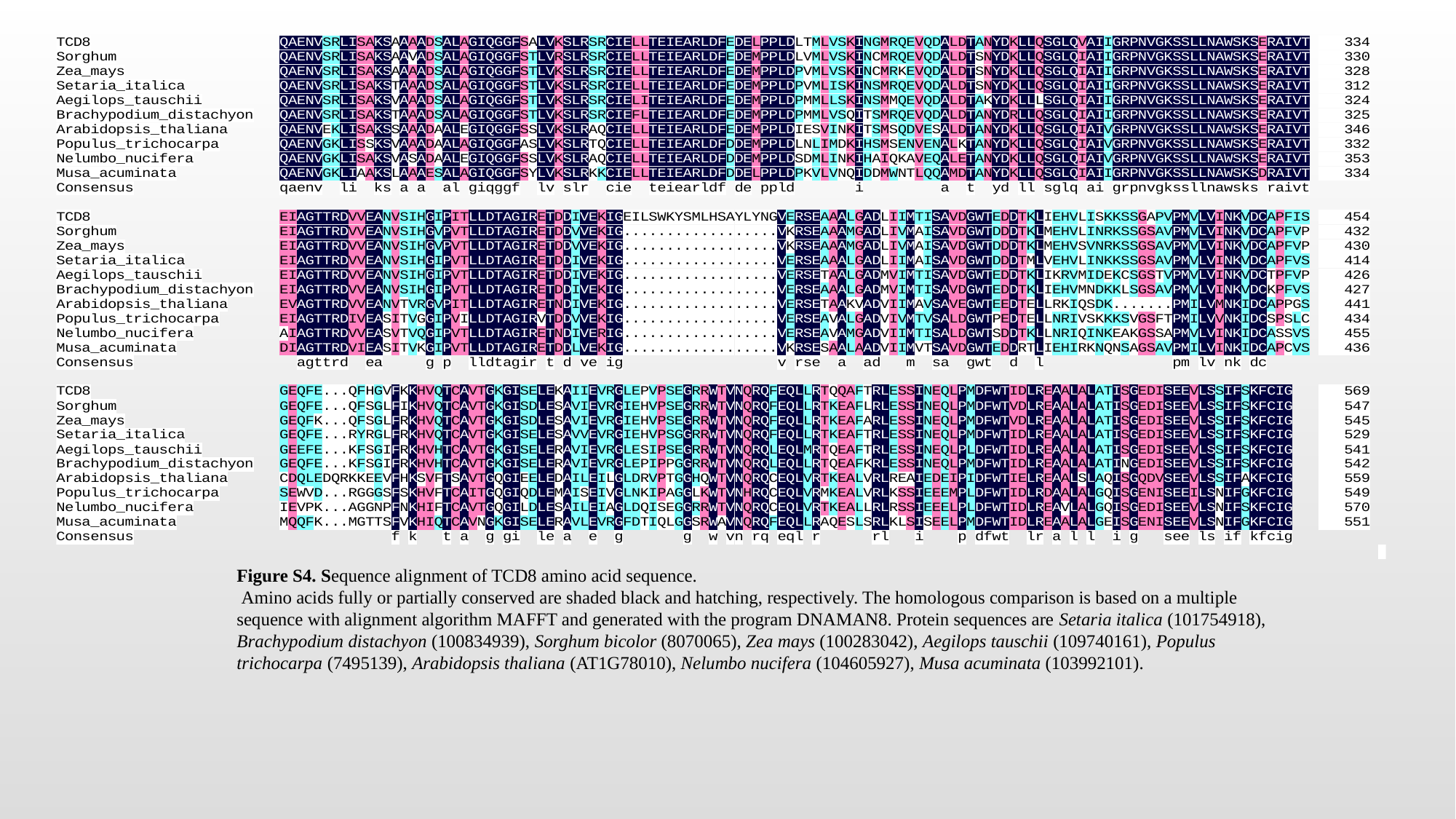

Figure S4. Sequence alignment of TCD8 amino acid sequence.
 Amino acids fully or partially conserved are shaded black and hatching, respectively. The homologous comparison is based on a multiple sequence with alignment algorithm MAFFT and generated with the program DNAMAN8. Protein sequences are Setaria italica (101754918), Brachypodium distachyon (100834939), Sorghum bicolor (8070065), Zea mays (100283042), Aegilops tauschii (109740161), Populus trichocarpa (7495139), Arabidopsis thaliana (AT1G78010), Nelumbo nucifera (104605927), Musa acuminata (103992101).

## Slide 5
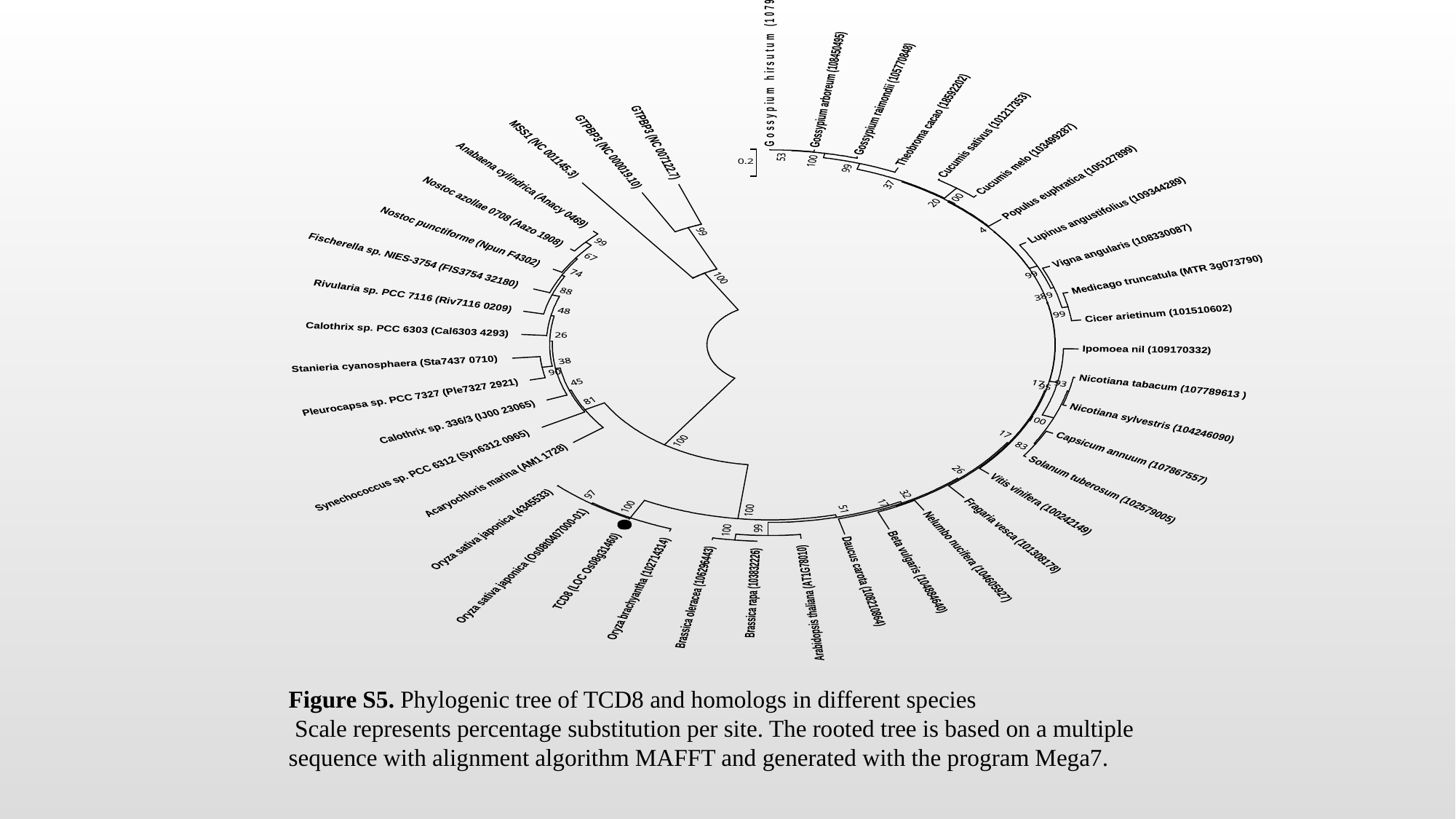

Figure S5. Phylogenic tree of TCD8 and homologs in different species
 Scale represents percentage substitution per site. The rooted tree is based on a multiple sequence with alignment algorithm MAFFT and generated with the program Mega7.

## Slide 6
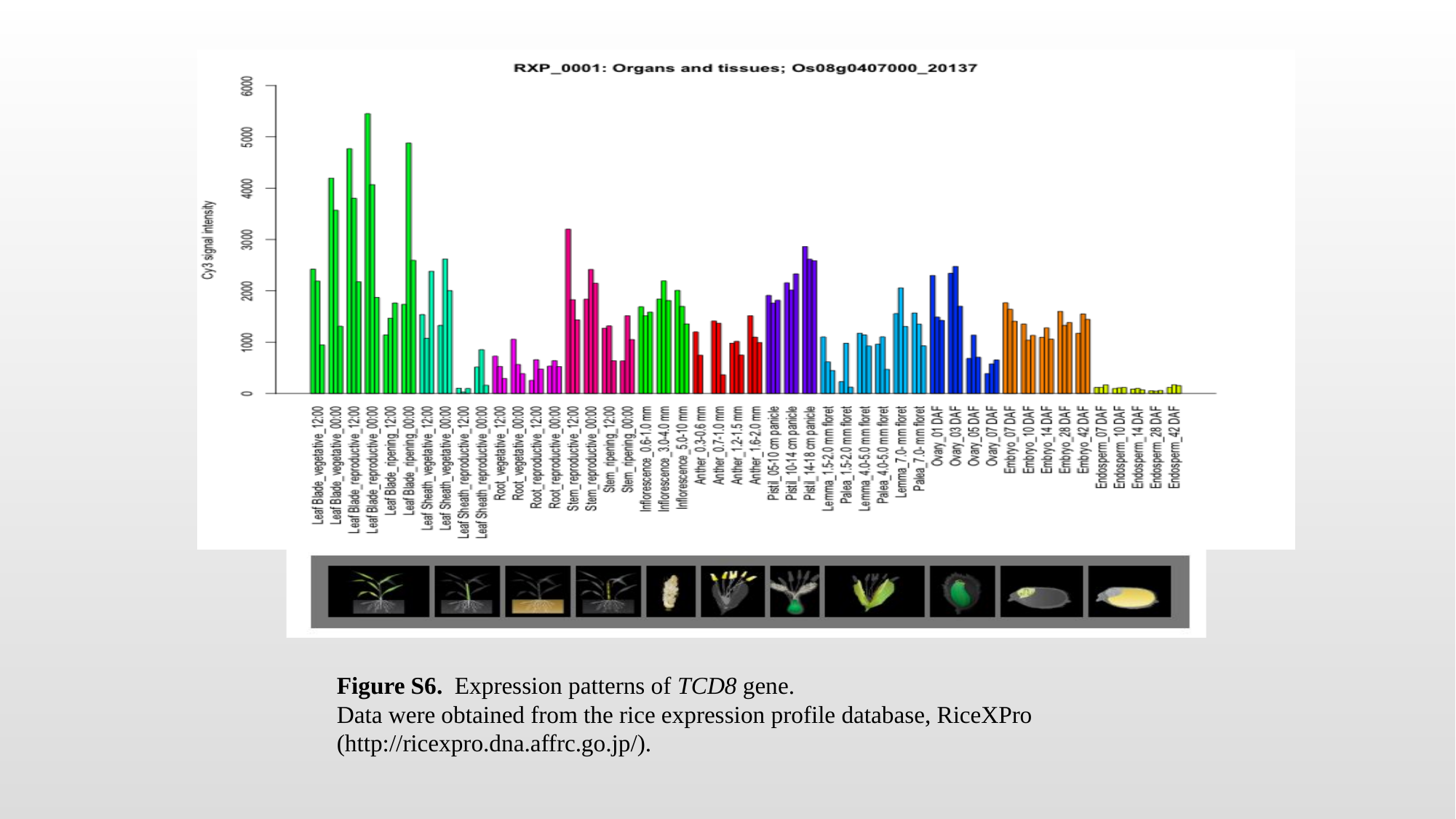

Figure S6. Expression patterns of TCD8 gene.
Data were obtained from the rice expression profile database, RiceXPro (http://ricexpro.dna.affrc.go.jp/).
